# Incidence of Glaucoma in Type 2 Diabetes Patients Treated With GLP‐1 Receptor Agonists: A Systematic Review and Meta‐Analysis

**DOI:** 10.1002/edm2.70059

**Published:** 2025-06-13

**Authors:** Maheen Asif, Aliza Asif, Ummi Aiman Rahman, Hanzala Ahmed Farooqi, Oshaz Fatima, Waqar Ali, Uzair Jafar, Mohammed Hammad Jaber

**Affiliations:** ^1^ Services Institute of Medical Sciences Lahore Pakistan; ^2^ Department of Endocrinolgy King Edward Medical University Lahore Pakistan; ^3^ Islamic International Medical College Riphah International University Rawalpindi Pakistan; ^4^ Faculty of Medicine Alzaiem Alazhari University Khartoum Sudan

**Keywords:** glaucoma, GLP‐1RA, meta analysis, T2DM

## Abstract

**Aims:**

Glaucoma, a leading cause of irreversible blindness, is particularly prevalent among individuals with Type 2 Diabetes Mellitus (T2DM), a known risk factor for the disease. This systematic review and meta‐analysis aimed to evaluate the incidence of glaucoma in T2DM patients treated with Glucagon‐Like Peptide 1 Receptor Agonists (GLP‐1RAs) compared to those using other antihyperglycaemic agents.

**Materials and Methods:**

A comprehensive search of literature was conducted using MEDLINE (PubMed), the Cochrane Library, Google Scholar, and Scopus up to September 14, 2024. Observational studies that reported the incidence of glaucoma among T2DM patients using GLP‐1RAs versus other antihyperglycaemic drugs were included. Data analysis employed the random‐effects model, presenting odds ratios (OR) with 95% confidence intervals (CI). Heterogeneity was assessed using *I* ^2^ statistics, and a sensitivity analysis was performed to test the result's robustness.

**Results:**

Five observational studies involving 2,500,430 participants met the inclusion criteria. The meta‐analysis indicated that GLP‐1RA use was associated with a nonsignificant reduction in the incidence of glaucoma (OR: 0.78; 95% CI: 0.60 to 1.02; *p* = 0.01: *I* ^2^ = 88%). Sensitivity analysis by leave‐one‐out method showed a significant reduction of glaucoma in GLP‐1 RA users.

**Conclusions:**

In conclusion, GLP‐1RA usage in T2DM patients may be beneficial in lowering the risk of glaucoma under some circumstances. These results advocate for further clinical studies to confirm GLP‐1RAs' protective ocular effects, potentially influencing future treatment guidelines and preventive care strategies for glaucoma patients.

## Graphical abstract

GLP‐1RA use in T2DM may reduce risk of glaucoma: A meta‐analysis and systematic review.

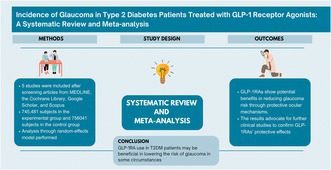

## 1 Introduction

Glaucoma is the leading cause of permanent blindness worldwide. Primary open‐angle glaucoma (POAG) is the most common type and is anticipated to impact around 112 million individuals globally by 2040 [1]. Current management strategies for glaucoma aim to reduce intraocular pressure (IOP). However, despite extensive therapy, 42% of patients are expected to become blind in one eye and 16% in both [2]. Glaucoma is marked by progressive optic neuropathy and is frequently associated with raised intraocular pressure (IOP), highlighting the necessity for prompt diagnosis and intervention [3]. The sequence of events includes optic nerve excavation and thinning of the retinal ganglion cell layer initially, with this period being asymptomatic in most cases. As glaucoma advances, the retinal ganglion cell layer further deteriorates due to extensive atrophy, and significant narrowing of the visual field occurs, resulting in considerable visual impairment [1, 2, 3, 4].

Despite comprehensive research, the pathogenesis, exact causes, and risk factors are multifaceted and not well understood [3]. Type 2 Diabetes Mellitus (T2DM) is a recognised risk factor for glaucoma. T2DM increases the risk of developing glaucoma subtypes such as POAG and neovascular glaucoma due to impaired vascular autoregulation of the retina and optic nerve, leading to microvascular damage, compromised neuronal and glial cell function resulting in neurodegeneration, and metabolic disturbances caused by chronic hyperglycaemic conditions [5, 6, 7]. Suboptimal glycaemic control has been linked to an increased risk of glaucoma onset, emphasising the necessity of regulating blood glucose levels in individuals with diabetes [7]. Current treatment regimens for T2DM primarily include the use of antidiabetic medications, with the addition of insulin in more severe scenarios [8]. Among these medicines are Glucagon‐Like Peptide 1 Receptor Agonists (GLP‐1RAs), first approved by the United States Food and Drug Administration (FDA) in 2005 as an antidiabetic medication, and then in 2014 for weight loss in non‐diabetic individuals [9]. GLP‐1RAs have been reported to reduce glycated Haemoglobin (HbA1c) levels by 2% [10].

In addition to its role in regulating glucose levels, GLP‐1RAs have protective effects on other organs and organ systems as well [10]. Their use has been shown to preserve renal function and play a role in treating chronic kidney disease in diabetic patients [11, 12, 13], along with a decline in the incidence of cardiovascular events, all‐cause mortality, and obesity [13, 14, 15]. Given these benefits, previous reports have demonstrated a significant increase in GLP‐1RA usage over the past decade [16], suggesting the importance of investigating whether these medications may also play a role in ocular complications of diabetes. In addition to this, T2DM patients using GLP‐1RAs are less likely to develop dry eye disease compared to those on other antidiabetic drugs like metformin [17, 18]. Previous research suggests that GLP‐1RAs can influence the course of various ocular conditions [19, 20]. Various mechanisms have been proposed for this effect. They act by decreasing neurodegenerative changes in the retina [20], mitigating microvascular damage, as well as preventing damage to the blood‐retinal barrier [21, 22]. Considering the vascular‐protective properties of GLP‐1RAs and the association between open‐angle glaucoma (OAG) and compromised ocular vasculature, a potential correlation between GLP‐1RA usage and the incidence of OAG may be present [23, 24].

While several observational studies have explored the potential association between reduced glaucoma incidence in T2DM patients using GLP‐1RAs compared to those on other antihyperglycaemic agents, there remains a significant gap in consolidating these findings. The absence of a comprehensive pooled analysis to accurately quantify the protective effect of GLP‐1RAs against glaucoma development in the T2DM population limits our understanding of the magnitude and consistency of this association. Addressing this gap is crucial for guiding clinical decisions, developing future treatment guidelines, and optimising therapeutic strategies. Therefore, we conducted this meta‐analysis to provide a robust, quantitative synthesis of available data and determine the pooled effect of GLP‐1RA use on glaucoma risk in patients with T2DM.

## 2 Methods

### 2.1 Search Strategy

This meta‐analysis was registered with PROSPERO (CRD42024600499) and carried out in accordance with the guidelines outlined in the Preferred Reporting Items for Systematic Reviews and Meta‐Analyses (PRISMA) [25]. Ethical approval was not required since the study was an analysis of published observational studies.

### 2.2 Data Sources and Searching

Two independent reviewers conducted a systematic evaluation of the following databases from their inception to 14th September 2024: MEDLINE (PubMed), the Cochrane Library, Google Scholar, and Scopus using a search strategy consisting of relevant keywords and Medical Subject Headings (MeSH). Additionally, we conducted a partial grey literature search and backward citation tracking using reference lists of relevant articles. The detailed search strategy is given in the Supporting Information.

### 2.3 Study Eligibility and Selection

Studies were included if they (1) were observational studies (cohorts, cross‐sectionals, or case–controls), (2) evaluated the incidence of glaucoma in patients using GLP‐1RAs compared with patients taking any other anti‐hyperglycaemic medications.

Studies were excluded if they (1) were not in English, (3) were animal studies, (2) were case reports, review articles, commentaries, or letters, (4) were not peer‐reviewed.

### 2.4 Data Extraction

Two researchers independently extracted data from all the included studies using a pre‐determined data sheet. The following data were extracted from each study: author name, year of publication, country, study design, characteristics of the participants, number of participants, and outcomes of interest. The numbers of observed events were extracted. Any discrepancies between the two reviewers were resolved by a senior author.

### 2.5 Quality Assessment

Two reviewers independently evaluated the potential for bias in each study using the Newcastle‐Ottawa Scale (NOS) [26]. This scale assigns ratings from 0 to 9 points. Index values 0–2 suggest low quality, 3–5 signal moderate quality, and 6–9 show high quality. The NOS evaluation for cohort studies included the following components: (1) Representativeness of the exposed cohort; (2) Selection of the unexposed cohort; (3) Confirmation of exposure; (4) Verification that the outcome of interest was not present at the initiation of the research; (5) Comparability; (6) Evaluation of outcomes; (7) Follow‐up duration; (8) Sufficiency of cohort follow‐up. The NOS assessment for case–control studies comprised the following elements: (1) Adequacy of case definition; (2) Representativeness of cases; (3) Selection of controls; (4) Definition of controls; (5) Comparability; (6) Determination of exposure; (7) Consistent technique of determination for cases and controls; (8) Non‐response rate.

### 2.6 Outcome Variables

This analysis was designed to study the incidence of glaucoma in patients using GLP‐1RAs compared with patients taking any other anti‐hyperglycaemic medications.

### 2.7 Statistical Analysis

The incidence of glaucoma was represented using forest plots with 95% confidence intervals. The data analysis was conducted with the RevMan software version 5.4 [27] using the random effects model. We used odds ratio (OR) with corresponding 95% confidence intervals (CIs) as the effect measures for the dichotomous variables. The evaluation of heterogeneity within the studies was performed using a 95% confidence interval and *I* ^2^ statistical analysis. Heterogeneity was considered significant if the *I* ^2^ value was > 50%. A sensitivity analysis was performed, excluding Eng et al. [28] which compared GLP‐1RAs to Sodium‐glucose cotransporter‐2 inhibitors (SGLT2i) only.

## 3 Results

### 3.1 Search Results

**FIGURE 1:**
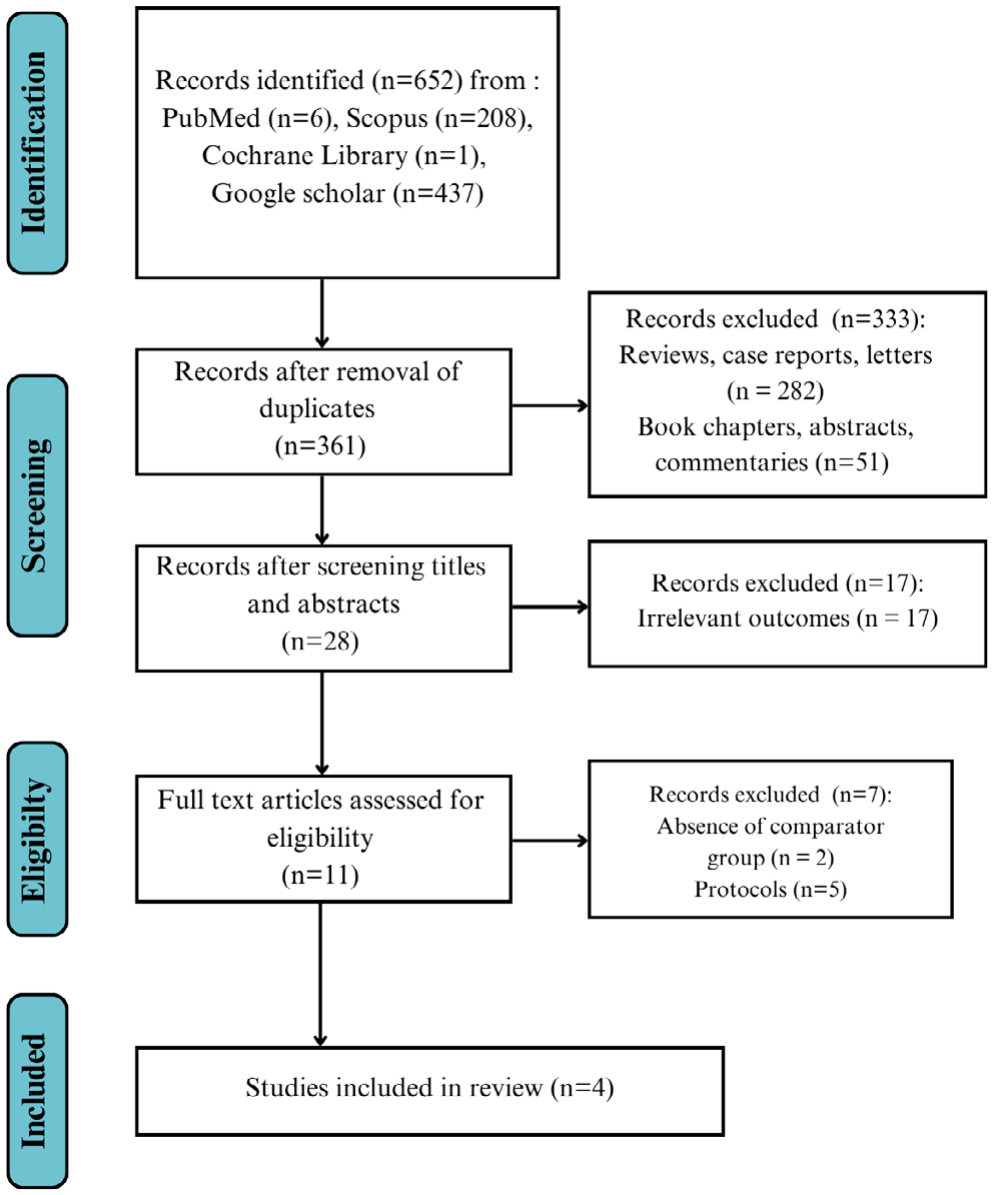
PRISMA flowchart of the study selection process.

Following the keyword search and after removing the duplicates, a total of 12 studies were subjected to full‐text screening. Two of them lacked a comparator group, and five of them were protocols. A total of five studies with 2,500,430 participants were included in our meta‐analysis [28, 29, 30, 31, 32]. One study was multinational, while the rest were in one country each. All studies compared GLP‐1RAs to all oral antihyperglycaemics except Eng et al., which compared GLP‐1RAs to SGLT2i alone. The detailed screening process is shown in Figure 1.

### 3.2 Study Characteristics

Table 1 presents a summary of the included study characteristics.

**TABLE 1:** Characteristics of included studies.

Study ID	Chuang et al. [29]	Niazi et al. [30]	Muayad et al. [31]	Sterling et al. [32]	Eng et al. (2024) [28]
Study setting	Taiwan	Denmark	Cambridge Morocco USA	USA	USA
Study design	Retrospective cohort study	Case‐control study	Retrospective cohort study	Retrospective cohort study	Cohort study
No. of participants	GLP‐1RA user: 1366 Control: 2732	GLP‐1 RA user: 1819 Control: 8603	GLP‐1 RA user: 61,998 Control: 61,998	GLP‐1 RA user: 1961 Control: 4371	GLP‐1 RA user: 804,561 Control: 864,397
Sex *n* (%)	*n* GLP‐1 RA user: Male: 770 Female: 596 Control: Male: 1540 Female: 1192	Male Case: 959 (55.2) Control: 4795 (55.2)	GLP‐1 RA user: Male: 21,387 (34.50) Female: 35,450 (57.18) Unknown: 5161 (8.32) Control: Male: 21,142 (34.10) Female: 35,852 (57.83) Unknown: 5004 (8.07)	GLP‐1 RA user: Female: 1028 (52.42) Male: 933 (47.58) Control: Female: 2271 (51.96) Male: 2100 (48.04)	Female *n* (%) 333,821 (46.2)
Age mean (SD)	*n* GLP‐1 RA user: 20–39: 428 40–49: 374 50–59: 366 60–69: 153 70–79: 36 ≥ 80: 9 Control: 20–39: 703 40–49: 852 50–59: 774 60–69: 345 70–79: 38 ≥ 80: 20	Case: 65.4 (10.6) Control: 65.2 (10.7)	GLP‐1 RA user: 56.1 (13.6) Control: 55.8 (15.5)	GLP‐1 RA user: 55.4 (10.4) Control: 55.6 (10.6)	59.1 (11.1)
Hypertension *n* (%)	NR	Case: 28 (1.6) Control: 178 (2.0)	GLP‐1 RA user: 41,696 (67.2) Control: 41,066 (66.2)	GLP‐1 RA user: 1722 (87.8) Control: 3507 (80.2)	547,254 (75.8)
Hyperlipidemia *n* (%)	NR	NR	GLP‐1 RA user: 30,222 (48.7) Control: 29,212 (47.1)	GLP‐1 RA user: 1792 (91.4) Control: 3735 (85.4)	523,443 (72.5)
Chronic kidney disease *n* (%)	NR	NR	GLP‐1 RA user: 9053 (14.6) Control: 8689 (14.0)	GLP‐1 RA user: 798 (40.7) Control: 1188 (27.2)	52,648 (7.3)

Abbreviations: GLP‐1 RA, Glucagon‐like peptide 1 receptor agonist; NR, not reported.

### 3.3 Risk of Bias in Included Studies

The summary of the quality of the studies, utilising the Newcastle‐Ottawa Scale (NOS), is presented in Tables 2 and 3. The quality of all the studies used in this meta‐analysis is high, as four of the studies have 9 stars [28, 29, 30, 31] and one of the studies has 8 stars [32].

**TABLE 2:** Quality assessment of cohort studies.

Sr. No.	Study	Selection				Comparability	Outcome			Total
		Representativeness of the exposed cohort	Selection of the non‐exposed cohort	Ascertainment of exposure	Outcome not present at start of study		Assessment of outcome	Length of follow‐up	Adequacy of follow‐up	
1	Muayad et al. [31]	*	*	*	*	**	*	*	*	9
2	Sterling et al. [32]	/	*	*	*	**	*	*	*	8
3	Chuang et al. [29]	*	*	*	*	**	*	*	*	9
4	Eng et al. [28]	*	*	*	*	**	*	*	*	9

*Note:* * refers to 1 score. ** refers to 2 score.

**TABLE 3:** Quality assessment of case–control study.

Sr. No.	Study	Selection				Comparability	Exposure			Total
		Is the case definition adequate	Representativeness of the cases	Selection of controls	Definition of controls		Ascertainment of exposure	Ascertainment method for cases and controls	Non‐response rate	
1	Niazi et al. [30]	*	*	*	*	**	*	*	*	9

*Note:* * refers to 1 score. ** refers to 2 score.

### 3.4 Data Synthesis and Meta‐Analysis

Five observational studies reported the incidence of glaucoma in GLP‐1RA users compared to other anti‐hyperglycaemic medications [28, 29, 30, 31, 32]. Table 4 provides a concise overview of the meta‐analyses conducted for the outcome.

**TABLE 4:** Outcome.

	Included studies	Participants	OR	95% CI	*p*	Heterogeneity		Model
						*I* ^2^	*P*_het_	
Glaucoma	5 [28, 29, 30, 31, 32]	2,500,430	0.78	0.60–1.02	0. 07	88%	< 0.00001	Random effect

### 3.5 Glaucoma Incidence

**FIGURE 2:**
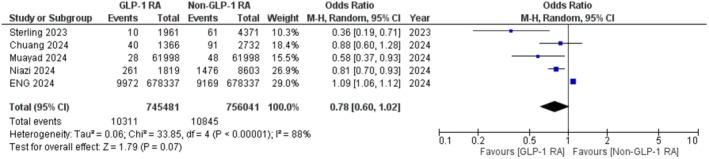
Forest plot of the incidence of glaucoma in GLP‐1 RA users compared to controls.

**FIGURE 3:**
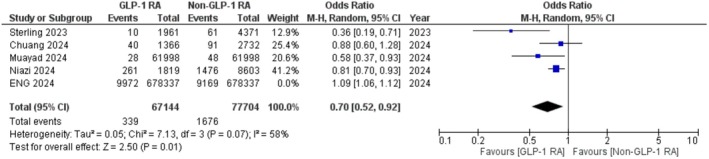
Sensitivity analysis for the incidence of glaucoma in GLP‐1 RA users.

Five studies [28, 29, 30, 31, 32] with 745,481 subjects in the experimental group and 756,041 subjects in the control group were analysed. The random effect model was used. Overall, GLP‐1RA use was associated with a nonsignificant reduction in the incidence of glaucoma (OR: 0.78; 95% CI: 0.60 to 1.02; *p* = 0.01; Figure 2). The heterogeneity was observed to be significant (*I* ^2^ = 88%). A sensitivity analysis was performed, excluding Eng et al. [28] which compared GLP‐1RAs to Sodium‐glucose cotransporter‐2 inhibitors (SGLT2i) only. Sensitivity analysis showed that GLP‐1RAs may be beneficial in reducing the incidence of glaucoma compared to other anti‐hyperglycaemics (OR: 0.70; 95% CI: 0.52 to 0.92; *p* = 0.01; Figure 3).

## 4 Discussion

Our systematic review and meta‐analysis revealed an overall comparable risk of glaucoma among type‐2 diabetic patients using GLP‐1RAs compared to those on other anti‐hyperglycaemic medications. Interestingly, when Eng et al. were excluded, we found a significantly lower incidence of glaucoma in type‐2 diabetic patients using GLP‐1RAs. Recent years have seen an increase in research on the benefits of GLP‐1RAs for general health, as well as ocular health, with a focus on glaucoma. Numerous investigations exploring these relationships have yielded information regarding the possible therapeutic applications of GLP‐1RAs in the management of systemic and ocular health.

Building on these general findings, we delved into specific studies to understand the nuanced effects of GLP‐1RAs on glaucoma incidence. Four of the included studies compared GLP‐1RAs to all anti‐hyperglycaemic agents, especially biguanides. The pooled results of these studies showed an overall reduced risk of glaucoma. Not surprisingly, Eng et al. 2024 [28] compared GLP‐1RAs to SGLT2i only and found different results. We did not have enough studies comparing GLP‐1RAs to SGLT2i to perform a subgroup analysis. Although the results may seem insignificant, the sensitivity analysis points toward the potential beneficial role of GLP‐1RAs in managing glaucoma compared with traditional anti‐hyperglycaemics.

The relationship between GLP‐1RAs and changes in intraocular pressure (IOP), a crucial component in the development of glaucoma, was examined in a noteworthy study by Hallaj et al. Comparing individuals on GLP‐1RAs to those on other antidiabetic drugs, their results showed that the former had a decreased probability of receiving a new glaucoma diagnosis. The study also revealed a statistically significant drop in IOP after starting GLP‐1RA treatment, especially in those with a history of glaucoma or pre‐existing excessive IOP [33]. Carris et al. conducted a study that included a thorough summary of the advantages of combining basal insulin with GLP‐1RAs in the management of type 2 diabetes. They pointed out that this combination of medications has wider health consequences, such as weight loss and possible cardiovascular advantages, in addition to improving glycaemic control. By treating risk factors frequently linked to diabetic problems, such as diabetic retinopathy and glaucoma, the systemic health benefits of GLP‐1RA medication, including reductions in blood pressure and improved lipid profiles, may indirectly improve ocular health [34]. Madsbad et al. noted the pleiotropic effects of GLP‐1RAs, which include reductions in cardiovascular risk variables and general metabolic health. These systemic advantages are important because they may help people with diabetes to experience fewer ocular problems. GLP‐1RAs have a variety of actions, including those that affect inflammation and insulin sensitivity, which makes them useful tools for managing diabetes and preventing eye disorders [35].

These neuroprotective benefits are closely associated with the anti‐inflammatory properties of GLP‐1RAs, illustrating how they collectively contribute to ocular health. According to Sterling et al., GLP‐1RAs have the potential to directly protect patients with glaucoma by lowering retinal inflammation and neuronal death linked to ocular hypertension [36]. Further evidence that GLP‐1 receptors are present in ocular tissues and may mediate local protective effects comes from the work of Lawrence et al., which shows that both topical and systemic treatment with GLP‐1RAs can rescue retinal ganglion cells in models of hypertensive glaucoma [37]. Moreover, Mouhammad et al. highlighted how GLP‐1RAs could revolutionise the treatment of glaucoma and urged clinical research to explore their neuroprotective qualities better [38].

Multiple underlying mechanisms explain the protective effects of GLP‐1RAs on ocular health, especially with regard to glaucoma. These mechanisms include enhancement of insulin sensitivity, reduction of inflammation, and promotion of neuroprotection. Understanding how GLP‐1RAs reduce the risk of glaucomatous damage requires an understanding of these pathways. Firstly, GLP‐1RAs have been shown to enhance insulin sensitivity, which is particularly relevant in the context of diabetes‐related ocular complications. According to Kim et al., individuals with type 2 diabetes mellitus who received basal insulin in addition to GLP‐1RA therapy experienced weight loss and improved glycaemic control. This was achieved by reducing the total insulin dose (T2DM) [39]. Given that insulin resistance and hyperglycaemia are established risk factors for the development of diabetic retinopathy and possibly glaucoma, this improvement in insulin sensitivity is noteworthy. GLP‐1RAs may significantly lower the incidence of diabetic eye problems by enhancing metabolic indices.

Nizari et al. showed that GLP‐1 receptor activation promotes neuroprotection against ischaemic damage. This suggests that GLP‐1RAs' anti‐inflammatory properties may be involved in shielding retinal neurons from glaucomatous damage [40]. As persistent inflammation is known to accelerate the development of glaucoma and other neurological illnesses, reducing inflammation is essential. Moreover, the neuroprotective effects of GLP‐1RAs are particularly noteworthy. Hernández et al. provided evidence that GLP‐1RAs activate the AKT signalling pathway, which is essential for neuronal survival. This pathway is critical for mediating the neuroprotective effects of GLP‐1RAs, as it promotes cell survival and reduces apoptosis in retinal neurons [19].

## 5 Limitations

The primary strength of this meta‐analysis is that, to date, it is the only analysis conducted on this topic. Our results should be interpreted with caution as only observational studies were included in our review, consisting of one case–control study and four cohort studies, with no randomised controlled trials (RCTs) available, indicating a substantial deficiency of robust data. Furthermore, our outcome exhibited considerable heterogeneity (*I* ^2^ = 88%). The observed heterogeneity could be due to the varying study designs, with one being a case–control study [30] and four being cohort studies [28, 29, 31, 32]. Furthermore, the follow‐up lengths varied; one study had a follow‐up of 12 years [30], another 11 years [32], one 6 years [29], and the remaining had 3 years of follow‐up [31]. The studies did not have uniform protocols, such as Eng et al. 2024 [28] comparing GLP‐1RAs to SGLT2i, while the rest compared them to all oral antihyperglycaemic agents. The unavailability of further data on direct comparison with SGLT2i limits our ability to comment on their relative effect in glaucoma, and Chuang et al. employed a combination of GLP‐1RAs with metformin, which might contribute to the observed variability [27]. Despite a comprehensive analysis of the methodologies, we could not ascertain the reasons for this. Finally, the potential for publishing bias may exist.

## 6 Future Implications

The findings of our review suggest promising implications for future glaucoma management in patients with diabetes. Continued research should focus on the long‐term effects of GLP‐1RAs on ocular health, particularly their neuroprotective mechanisms and their influence on intraocular pressure. Large‐scale clinical trials with uniform protocols exploring the combined use of GLP‐1RAs with other therapies could enhance the treatment protocols for diabetes‐related ocular conditions. There is a need for RCTs to make one‐to‐one comparisons between GLP‐1RAs and SGLT2i to better understand their comparative efficacy in preventing the incidence of glaucoma. Understanding the molecular pathways involved in the protective effects of GLP‐1RAs may lead to the development of targeted therapies aimed at preventing glaucoma. Expanding awareness among healthcare providers about these benefits is essential for improving patient outcomes. Even though we may not have enough data and literature to be able to modify the guidelines, the results point toward a potential benefit of GLP‐1RAs in diabetes‐related glaucoma. For this reason, it is necessary to combine these results and identify an overall trend so that such data are not lost, and further larger‐scale trials are encouraged, which can then provide enough evidence to guide therapies.

## 7 Conclusion

In conclusion, GLP‐1RA use in T2DM patients may be beneficial in lowering the risk of glaucoma in some circumstances. Several mechanisms, such as direct neuroprotective effects, decreased inflammation, and enhanced insulin sensitivity, contribute to the protective effects of GLP‐1RAs on ocular health. These results advocate for further clinical studies to confirm GLP‐1RAs' protective ocular effects, potentially influencing future treatment guidelines and preventive care strategies for patients with glaucoma.

## Author Contributions

**Maheen Asif:** conceptualization; literature search; data curation; formal analysis; investigation; methodology; software; validation; visualisation; writing – original and revised draft. **Aliza Asif:** conceptualization; literature search; data curation; formal analysis; investigation; methodology; software; validation; visualisation; writing – original and revised draft. **Ummi Aiman Rahman:** data curation; investigation; methodology; writing – original draft. **Hanzala Ahmed Farooqi:** data curation; formal analysis; methodology; writing – original draft. **Oshaz Fatima:** investigation; methodology; validation; visualisation; writing – revised draft. **Waqar Ali:** revised literature search; formal analysis; supervision; validation; writing – revised draft. **Uzair Jafar:** revised literature search; formal analysis; supervision; validation; writing – revised draft. **Mohammed Hammad Jaber:** conceptualization; investigation; methodology; resources; visualisation; writing – original draft.

## Ethics Statement

The authors have nothing to report.

## Consent

The authors have nothing to report.

## Conflicts of Interest

The authors declare no conflicts of interest.

## Supporting information


Data S1.

## Data Availability

The data supporting this study's findings are available from the corresponding author upon reasonable request.
