# Supplementary material for: Incidence of Glaucoma in Type 2 Diabetes Patients Treated With GLP‐1 Receptor Agonists: A Systematic Review and Meta‐Analysis
Source: Endocrinol Diabetes Metab. 2025 Jun 13;8(4):e70059. doi: 10.1002/edm2.70059 (PMC12163345; doi:10.1002/edm2.70059)
Supplement: Supplementary file 1 — Data S1. [file EDM2-8-e70059-s001.docx]

**PubMed**

| **Search number** | **Query** | **Results** |
| --- | --- | --- |
| #1 | "Diabetes mellitus"[All Fields] OR "type 2 diabetes mellitus"[All Fields] OR "diabetes"[All Fields] OR "T2DM"[All Fields] | 920070 |
| #2 | "GLP-1 receptor agonists"[All Fields] OR "glucagon-like peptide-1 receptor agonist"[All Fields] | 4097 |
| #3 | "glaucoma"[All Fields] | 87977 |
| #4 | "prospective"[All Fields] OR "prospective"[All Fields] OR "longitudinal"[All Fields] OR "cohort"[All Fields] OR "cohorts"[All Fields] OR "observational"[All Fields] OR "follow-up"[All Fields] OR "case-control"[All Fields] OR "cross-sectional"[All Fields] OR "population-based"[All Fields] OR "Prospective Studies"[All Fields] OR "Case-Control Studies"[All Fields] OR "Epidemiological Studies"[All Fields] OR "Longitudinal Studies"[All Fields] OR "Observational Study"[All Fields] | 4455703 |
| #5 | #1 AND #2 AND #3 AND #4 | 6 |

**Google Scholar**

| **Query** | **Results** |
| --- | --- |
| ("Diabetes mellitus" OR "type 2 diabetes mellitus" OR "T2DM") AND ("GLP-1 receptor agonists" OR "glucagon-like peptide-1 receptor agonist") AND ("glaucoma") AND ("prospective" OR "prospective" OR "longitudinal" OR "cohort" OR "cohorts" OR "observational" OR "follow-up" OR "case-control" OR "cross-sectional" OR "population-based" OR "Prospective Studies" OR "Case-Control Studies" OR "Epidemiological Studies" OR "Longitudinal Studies" OR "Observational Study") | 437 |

**Scopus**

| **Query** | **Results** |
| --- | --- |
| TITLE-ABS-KEY ( (Diabetes mellitus OR type 2 diabetes mellitus OR T2DM) AND (GLP-1 receptor agonists OR glucagon-like peptide-1 receptor agonist) AND (glaucoma) AND ( prospective OR prospectively OR longitudinal OR cohort OR cohorts OR observational OR follow-up OR nested OR case-control OR cross-sectional ) ) | 208 |

**Cochrane**

| **Search**  **number** | **Query** | **Results** |
| --- | --- | --- |
| #1 | (Diabetes mellitus OR type 2 diabetes mellitus OR T2DM) | 87631 |
| #2 | (GLP-1 receptor agonists OR glucagon-like peptide-1 receptor agonist) | 5177 |
| #3 | (glaucoma) | 9477 |
| #4 | (prospective OR prospectively OR longitudinal OR cohort OR cohorts OR observational OR follow-up OR nested OR case-control OR cross-sectional) | 652276 |
| #5 | #1 AND #2 AND #3 | 1 |

|  | **Study removed** | **Heterogeneity before sensitivity analysis** | | **Heterogeneity after sensitivity analysis** | | ***p*-value  before sensitivity analysis** | ***p*-value  after sensitivity analysis** |
| --- | --- | --- | --- | --- | --- | --- | --- |
|  |  | ***p*-value** | ***I^2^*** | ***p*-value** | ***I^2^*** |  |  |
| Glaucoma | Chuang 2024 | <0.00001 | 88% | <0.00001 | 91% | 0.07 | 0.08 |
|  | Niazi 2024 |  |  | 0.0004 | 84% |  | 0.15 |
|  | Muayad 2024 |  |  | <0.00001 | 89% |  | 0.19 |
|  | Sterling 2023 |  |  | <0.0001 | 87% |  | 0.25 |
|  | Eng 2024 |  |  | 0.07 | 58% |  | 0.01 |

**S1**: Sensitivity analysis by leave-one-out method
